# Paddle Your Own Canoe

**Published:** 1869-05

**Authors:** 


### PADDLE YOUR OWN CANOE.

JUDGE S. gave his son a thousand dollars, telling him to go to college and graduate; the son returned at the end of the Freshman year without a dollar and with several ugly habits. About the close of the vacation, the Judge said to his son, "Well, William, are you going to college this year?"

"Have no money, father."

"But I gave you a thousand dollars to graduate on."

"It 's all gone, father."

"Very well, my son; it was all I could give you; you can't stay here; you must now pay your own way in the world."

A new light broke in upon the vision of the astonished young man. He accommodated himself to the situation; left home, made his way to college, graduated at the head of his class, studied law, became Governor of the State of New York, entered the Cabinet of the President of the United States, and has made a record for himself that will not soon die, being none other than WILLIAM H. SEWARD.

"I want no more money," said a gentleman the other day, who had retired from business, on the application of a friend to join in what promised to be a profitable investment. In answer to a look of surprise he continued, "I have three sons; I gave them all a classical education. One became a merchant, another a lawyer, a third a physician. I gave them all a fair start and they have come home to live on me. The doctor had no patients; the lawyer no clients, and the merchant no customers. They say to me, 'Father, it 's no use for us to work; you have a plenty, and we will have more than we can spend,—why should we be the slaves of business?' Why, now, should I want more money; what I have has made my sons useless to themselves, useless to society and to the world. Had they been compelled, as I was, to start out in life on nothing, and 'paddle their own canoe,' they might have been a credit to themselves and to me, instead of being as they are now, a disgrace to my family name."

The more a thriftless child is helped, the more he looks for it, and the more he has to be helped ever after; as it is in the moral world, so it is in the physical as pertaining to the health of our bodies, and millions die prematurely from not recognizing this principle in reference to bodily habits and functions.

The more nature is helped the more she has to be helped. If a stimulus is taken at a given hour every day it will not be a week before there will be felt a want of that stimulus about the regular hour, and very decided bodily discomfort follows if the artificial want is not satisfied. In this way millions are made abject slaves for life to the use of tobacco, tea, coffee, spirits, and opium. Unhealthful indulgences come upon us in the same way. If one goes to sleep to day at any hour and is not specially busy to-morrow he will feel inclined to take a nap, and soon he cannot do without it with any convenience.

If in medicine a tonic is taken for a few day to increase the appetite, or digestion, the system seems to look for it, and as all tonics contain alcohol, multitudes become, in effect, drunkards, before they are aware of it. If any medicine is taken to regulate the digestion, that medicine soon becomes necessary to that regulation, and the man is doomed to make an apothecary's shop of himself for the remainder of his life.

If the nostrils are dry and you snuff water up into them for this or any other cause, nature soon ceases to prepare the proper lubricant, and a troublesome habit is soon formed, which sometimes drives the disease down into the lungs.

If the hair is greased to make it look glossy for a specific occasion, in a short time it must be repeated; this grease runs down to the root of the hair or scalp and carries with it the dust which is always falling on the head, soon forming a cement which closes the pores of the scalp, prevents the healthful flow of the fluids which are intended to keep each hair healthful, pliant and soft—leaves it to become dry, harsh, imperfectly nourished, dead. The tons of beautiful hair now worn, grew on the heads of the young girls of Normandy who keep their hair covered with a handkerchief and by no possibility ever allow any thing to be put on it, not even water. It is vigorous health with some constitutional aid which gives them their wealth of hair, requiring no dressing whatever. We destroy it by torturing it; straining it against its natural direction, curling it, frizzling it, and keeping a bed of grease and dust around its roots which impedes the flow of natural nourishment to it, causes it to die and fall out before our daughters have reached womanhood, and too often before they have left their teens.

In the preservation of our health let us rely more on nature; take time first, and if that does not do, consult a physician; but as for taking medicine for every little trifling thing to help us into health—throw nature on her own resources, let her help herself. And as for your children, let them feel, however much money you have, that it will pass to them only until they have shown that they can help themselves—"can paddle their own canoe."

